# Epstein–Barr virus peptides derived from latent cycle proteins alter NKG2A + NK cell effector function

**DOI:** 10.1038/s41598-020-76344-3

**Published:** 2020-11-17

**Authors:** Berenice Mbiribindi, Josselyn K. Pena, Matthew P. Arvedson, Claudia Moreno Romero, Sarah R. McCarthy, Olivia L. Hatton, Carlos O. Esquivel, Olivia M. Martinez, Sheri M. Krams

**Affiliations:** 1 Division of Abdominal Transplantation, Department of Surgery, Stanford University School of Medicine, Stanford, CA USA; 2 Department of Molecular Biology, Colorado College, Colorado Springs, CO USA

**Keywords:** Cell biology, Immunology

## Abstract

Natural killer (NK) cells control viral infection through the interaction between inhibitory receptors and human leukocyte antigen (HLA) ligands and bound peptide. NK cells expressing the inhibitory receptor NKG2A/CD94 recognize and respond to autologous B cells latently infected with Epstein–Barr virus (EBV). The mechanism is not yet understood, thus we investigated peptides derived from seven latent proteins of EBV in the interaction of NKG2A and its ligand HLA-E. Functional analysis demonstrated that EBV peptides can bind to HLA-E and block inhibition of NK cell effector function. Moreover, analysis of DNA from 79 subjects showed sequence variations in the latent protein, LMP1, which alters NK responses to EBV. We provide evidence that peptides derived from EBV latent cycle proteins can impair the recognition of NKG2A despite being presented by HLA-E, resulting in NK cell activation.

## Introduction

Epstein–Barr virus (EBV) is a ubiquitous γ-herpesvirus that persists as a chronic, asymptomatic infection in over 90% of the adult human population. EBV infections in young children are mainly asymptomatic and the infection subsides due to a vigorous host T cell response with the virus subsequently transitioning to latency in a subset of memory B cells^1,2^. In adolescents, EBV infection can manifest as glandular fever, lymphadenopathy, and a sore throat, termed infectious mononucleosis (IM)^3^. IM is associated with a transient proliferation of EBV-infected B cells and is typically self-limiting with the virus ultimately persisting into a latent phase in infected B cells. The latency phase of EBV infection is marked by the expression of a specific set of latent cycle proteins leading to 3 distinct types corresponding to latency I, II and III. Type I latency is limited to the expression of Epstein–Barr virus nuclear antigen 1 (EBNA-1). EBNA-1 is a critical regulator of transcription of host cell genes and enhances survival of latently infected cells^4^. In addition to EBNA-1, type II latency also includes the expression of latent membrane protein 1 and 2 (LMP1 and LMP2). LMP1 and LMP2 provide relevant signals to promote the survival of the infected B cell. LMP2 mimics B cell receptor (BCR) signaling while LMP1 mimics CD40 signaling^5^. Additionally, both LMP1 and LMP2 jointly contribute to oncogenic mechanisms by modulating DNA repair^6^. Type III latency is characterized by the co-expression of EBNA-1, EBNA-2, EBNA-3ABC, LMP1, LMP2A/B, EBNA-LP. EBNA-2 is first expressed after infection and has a crucial role in virus-mediated transformation. EBNA-3 proteins demonstrate redundant biological roles, and genetic studies using recombinant viruses show that only EBNA-3A and -3C, but not -3B, are essential for B-cell transformation in vitro^7–10^*.* EBNA-3A has been demonstrated to play a role in the regulation of cell survival in B cells immortalized by EBV^11^. Together, EBV proteins provide a variety of functions in the infection, replication, transformation, growth, and survival of infected cells^12,13^.

Failure to control latent EBV infection can result in a variety of EBV-associated malignancies, including lymphoproliferative diseases (EBV-LPD)^14^, particularly in immune-suppressed or immune-deficient individuals^15–17^. Several lines of evidence suggest that innate immune responses including natural killer (NK) cells are critical in host defense against EBV. NK cells play an important role in protection against viruses and tumor growth. Numerous studies in both animals and humans suggest that NK cells are critical in the host defense against EBV. It has been demonstrated that NK cell depletion in humanized mouse models correlates with exacerbated infectious mononucleosis ( IM ) and favors EBV-associated tumorigenesis^18,19^. Additionally, in vitro studies clearly have shown killing of EBV infected B cells by primary human NK cells^20,21^. During IM, NK cells eliminate infected B cells and augment the antigen-specific T cell response via release of immunomodulatory cytokines^22,23^ and NK cell deficiency leads to severe complications. Patients with X-linked lymphoproliferative syndrome and X-linked immunodeficiency with Mg2 + defect or neoplasia (XMEN) have NK deficiencies and suffer life-threatening complications of EBV infection^24^. Thus, NK cells are regarded as critical in the early immune response to EBV primary infection, but their role in controlling expansion of latently infected B cells is not yet clear.

NK cells display a heterogenous group of activating and inhibitory receptors on their cell surface which regulate effector function, central to which are the Killer Ig-like Receptors (KIR) as well as the C-lectin-like receptors (NKG2A, -C and -D)^25,26^. Previous studies from our group and others demonstrated that NK cells expressing NKG2A respond to autologous latently-infected B cells^27^ and proliferate when cultured with EBV-infected B cells^28^, supporting a role for NK cells in the response to latent EBV. NKG2A dimerizes with CD94 and recognizes the non-classical class I major histocompatibility complex (MHC-I) molecule human leukocyte antigen (HLA)-E^29^. Contrary to classical MHC-I molecules, HLA-E displays limited polymorphism. To date only two alleles are described as functionally relevant. The peptide-binding groove of HLA-E is usually occupied by nonameric peptides derived from the signal sequence of certain HLA-A, -B, -C, and -G molecules^30^. Here, we combined in silico analysis of HLA-E binding peptides from EBV with experiments using a reductionist model and we demonstrated that peptides derived from EBV latent cycle proteins can be presented by HLA-E and alter NKG2A + NK cell functions.

## Results

### In silico analysis of EBV peptides

**Figure 1:**
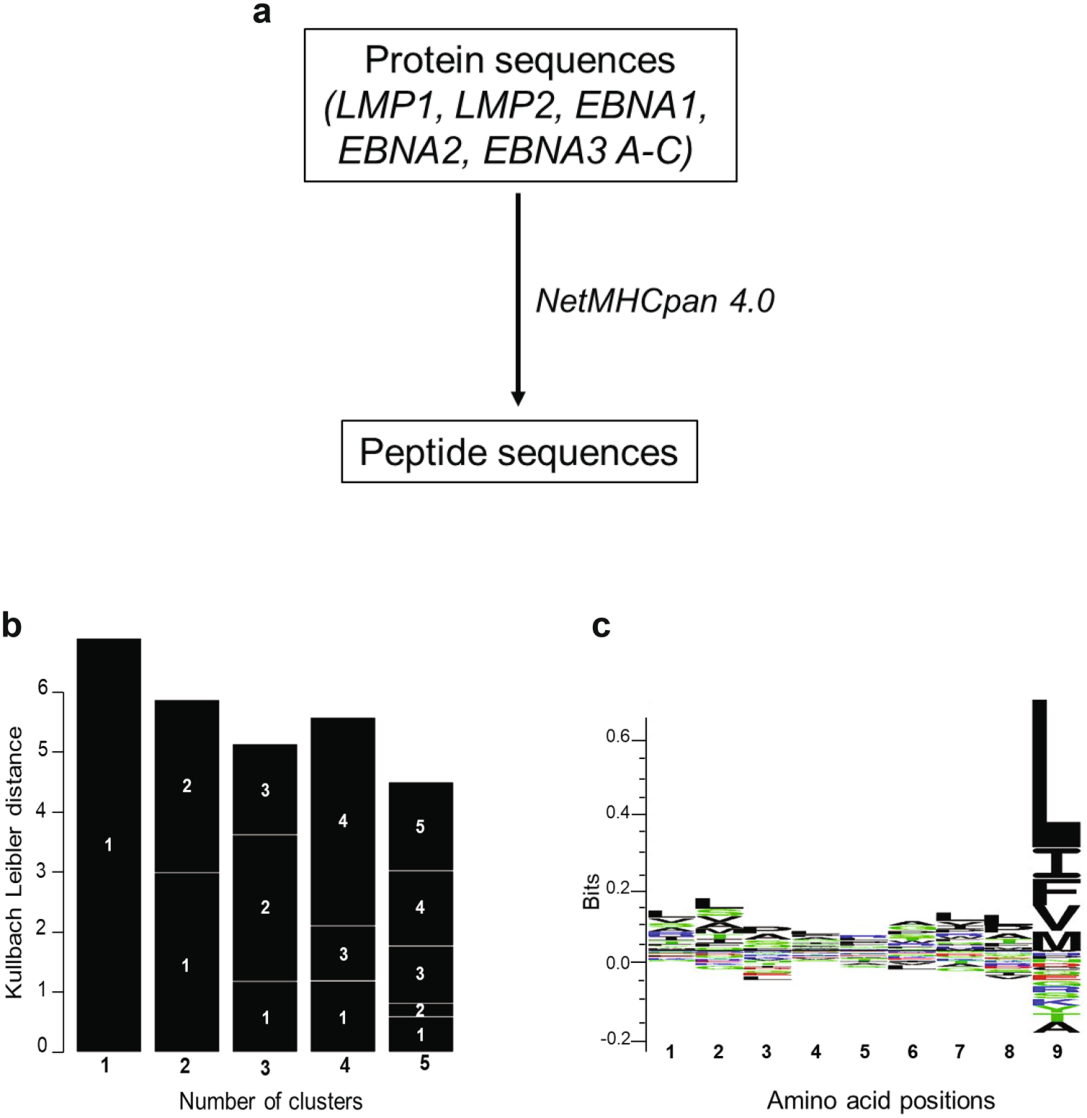
In silico analysis reveals HLA-E binding peptides derived from EBV latent cycle proteins. (**a**) Peptide sequence identification pipeline. (**b**) Gibbs clustering and Sequence logo of HLA-E peptides binders using the Gibb Cluster method. (**c**) Results are displayed in a Seq2Logo. At each position symbols represent the amino acids. Large symbols represent frequently observed amino acids, large stacks represent conserved positions and small stacks represents variable positions. Seq2Logo shows less variability at P9.

Previous studies have demonstrated that NKG2A + NK cells, but not NKG2C + NK cells, respond to B cells latently infected with EBV. NKG2A is an inhibitory receptor which normally prevents NK cell effector function when bound to HLA-E. To determine if peptides from the latent proteins of EBV bind to HLA-E*0101 allele, we used the UniProt database and NetMHCpan server pipeline to identify peptides from EBV-latent proteins (LMP1, LMP2, EBNA1, EBNA 2 and EBNA 3A-C) while taking into account endoplasmic reticulum (ER) processing when predicting peptides (Fig. 1a and Figures S1 a-b). This computational analysis of latent cycle proteins generated 61 peptides with the potential to bind to HLA-E (Fig. S1b). Subsequent alignment using GibbCluster demonstrated a distinct sequence motif (Figs. 1b,c). This analysis clearly showed that most of the sequences (n = 50) have a leucine (L) at position 9 (p9), the HLA-E main anchor residue^31^. Results obtained from the in silico experiments suggest that EBV latent proteins encode for peptides that could bind to and be presented by HLA-E*0101.

### Peptides derived from EBV latent proteins bind to HLA-E

**Figure 2:**
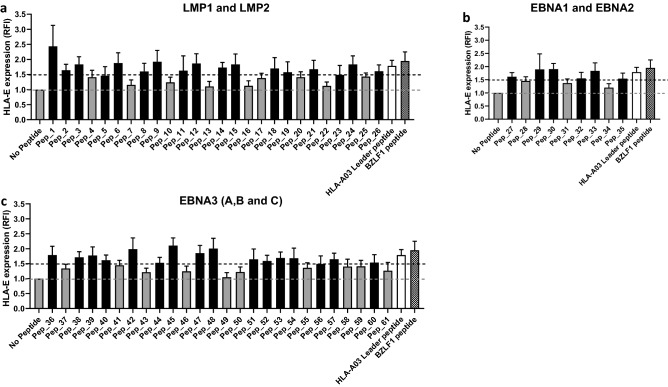
EBV derived peptides upregulate HLA-E surface expression. (**a**) LMP1 and LMP2, (**b**) EBNA-1 and ENBA-2, (**c**) EBNA-3A, -3B and -3C encoding peptides were pulsed onto target cells. HLA-E expression was assessed the following day. Peptides that bound HLA-E induced upregulation of HLA-E (mean fluorescence intensity (MFI) values) surface expression, which is shown as relative mean fluorescence intensity (RFI) as compared to HLA-E expression in absence of peptide (No Peptide). Endogenous peptide, HLA-A03 leader peptide (white filled bar) and viral peptide, BZLF1 (checkered bars) derived from EBV were used as positive controls. The dashed grey line represents the background expression in absence of peptide (cut-off set by No Peptide) and the black dashed line represents the cut-off for the peptides considered HLA-E binders. Peptides represented by black bars were considered as binders and peptides in grey bars non-binders. Mean ± SEM of two independent experiments.

TAP-deficient 721.174 cells do not express the TAP proteins required for peptide loading onto the MHC-I molecules^32,33^. Therefore, 721.174 cells have a markedly low level of MHC-I at their cell surface, which can be upregulated by exogenous peptide loading. We utilized this characteristic to determine the extent to which peptides from EBV latent proteins could bind to HLA-E and stabilize its surface expression on 721.174 cells. For each of the 61 peptides identified in our in silico analysis, we pulsed target cells with 200 µM peptide and HLA-E surface expression was quantified by flow cytometry. HLA-E binders (Fig. 2, black bars, and Fig. S2) were defined by having an MFI greater than the mean value plus 1.5 standard deviations of the “No Peptide” condition. Two peptides that have been previously shown to bind HLA-E were used as a positive control for HLA-E binding: the HLA-A03 leader peptide (white filled bar)^34^ and the BZLF1 peptide from an EBV lytic protein (checkered bar)^35^ (Fig. 2a–c). Seventeen peptides encoded by LMP1 and LMP2 (Fig. 2a), six peptides from EBNA1 and EBNA2 (Fig. 2b), and sixteen peptides from EBNA-3A, and -3C (Fig. 2c) bound to HLA-E. All peptides were titrated over a range of 0–400 µM, a subset of the peptides is shown in Fig. S2d. Taken together, we identified thirty-nine EBV peptides from latent cycle proteins that can bind to HLA-E and stabilize surface expression.

### Peptides from EBV latent proteins affect NK cell function

**Figure 3:**
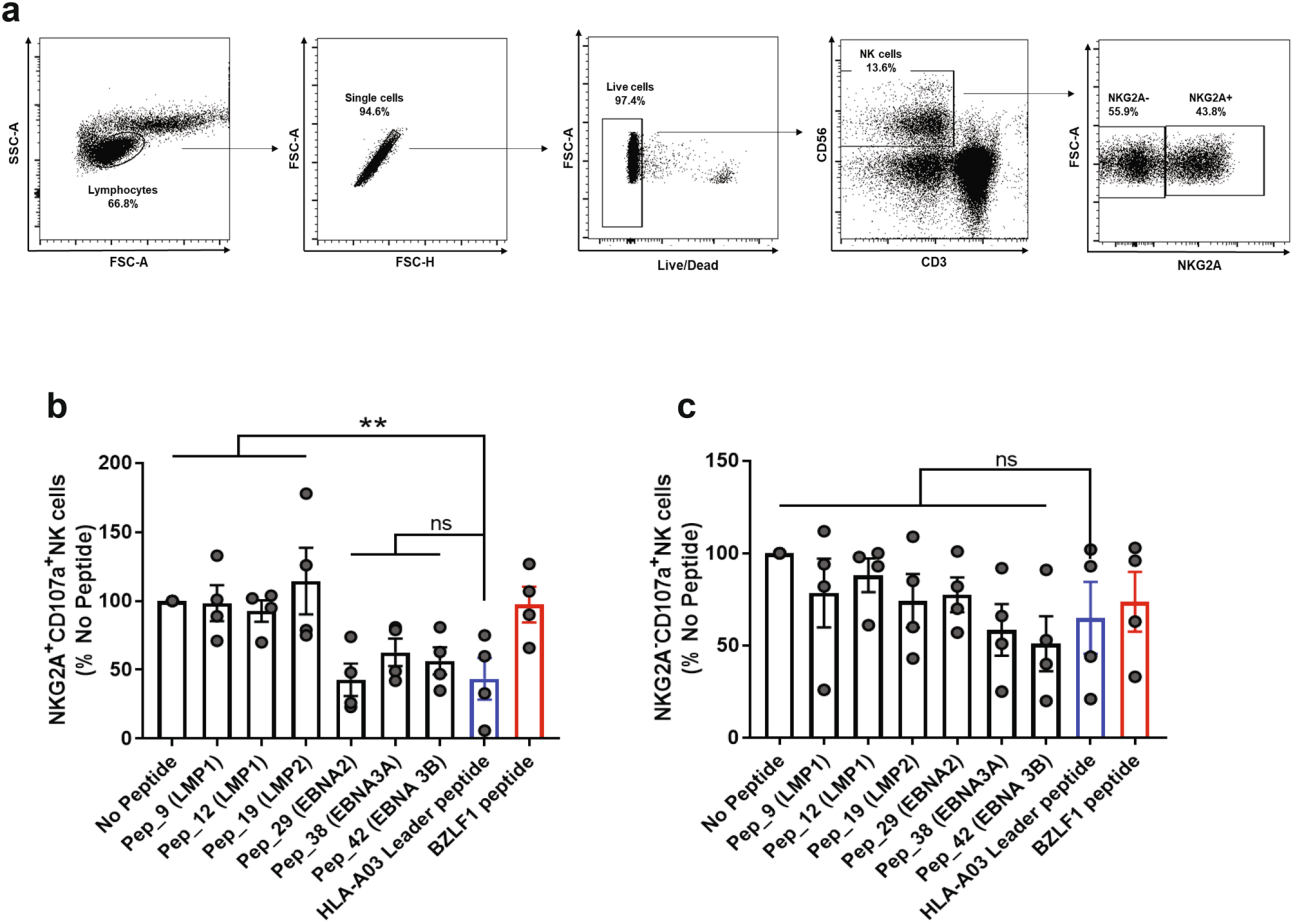
EBV peptides trigger NKG2A + NK cell degranulation. (**a**) Gating strategy for the identification of NKG2A + and NKG2A−NK cells within PBMC. (**b**) Comparison of NKG2A-positive NK cell degranulation against target cells loaded with EBV peptides. Peptides were individually tested using 721.174 cells. HLA-A03 leader peptide and BZLF1 peptides were respectively used as controls for inhibition and no inhibition. All values have been normalized to “No Peptide”. One-way ANOVA showed significance when compared the leader peptide condition with the others, p < 0.05. (**c**) Comparison of NKG2A-NK cell degranulation against target cells loaded with EBV peptides. The same controls were used as in panel A and all values have been normalized to “No Peptide”. No significant differences were observed between the different conditions. Data shown is representative of 4 donors.

To determine if peptides from EBV latent proteins can alter NKG2A + NK cell function, we performed NK cell degranulation assays focusing only on those peptides that bound to HLA-E. Donor PBMC were isolated and co-cultured with peptide-pulsed 721.174 target cells at an effector:target (E:T) ratio of 5:1. NKG2A + NK cell degranulation marked by CD107a expression was assessed by flow cytometry. After gating on lymphocytes, singlets and live cells, NK cells were identified as the CD56 + CD3-population (Fig. 3a). The HLA-A03 leader peptide (blue outlined bar) and BZLF1 peptide (red outlined bar) resulted in low (inhibition) and high (no inhibition) NKG2A + NK cell degranulation, respectively (Figs. 3b,c). Each individual peptide from our panel was investigated (Fig. S3a). Our data showed a subset of non-inhibitory peptides that significantly increased degranulation of NKG2A + NK cells when compared to the leader peptide control (blue outlined bar) and were mainly derived from LMP1 and LMP2 proteins (Fig. 3b and Fig. S3a). A second subset of peptides did inhibit NK cell function similar to the leader peptide control and were mainly derived from EBNA proteins (Fig. 3b and Fig. S3a). The function of NKG2A- NK cells, however, was not significantly altered by these peptides (Fig. 3c and Fig. S3b). Additional studies were performed with primary NK cells and their autologous lymphoblastoid cell line (LCL) (Fig. S4). HLA-E expression was poorly stabilized in autologous LCL likely due to high basal HLA-E expression (Fig. S4a). As a result, NK cell degranulation was not altered by the presence of the peptides presumably due to this lack of robust presentation by HLA-E (Fig. S4b-d).

These results suggest that some EBV latent peptides did alter NKG2A + NK cell inhibition against peptide-pulsed target cells. We determined that peptides encoded by LMPs tended to favor NK cell degranulation while peptides from EBNAs were inhibitory.

### Naturally occurring sequence variations of LMP1 protein prevent NKG2A + NK cell inhibition

**Figure 4:**
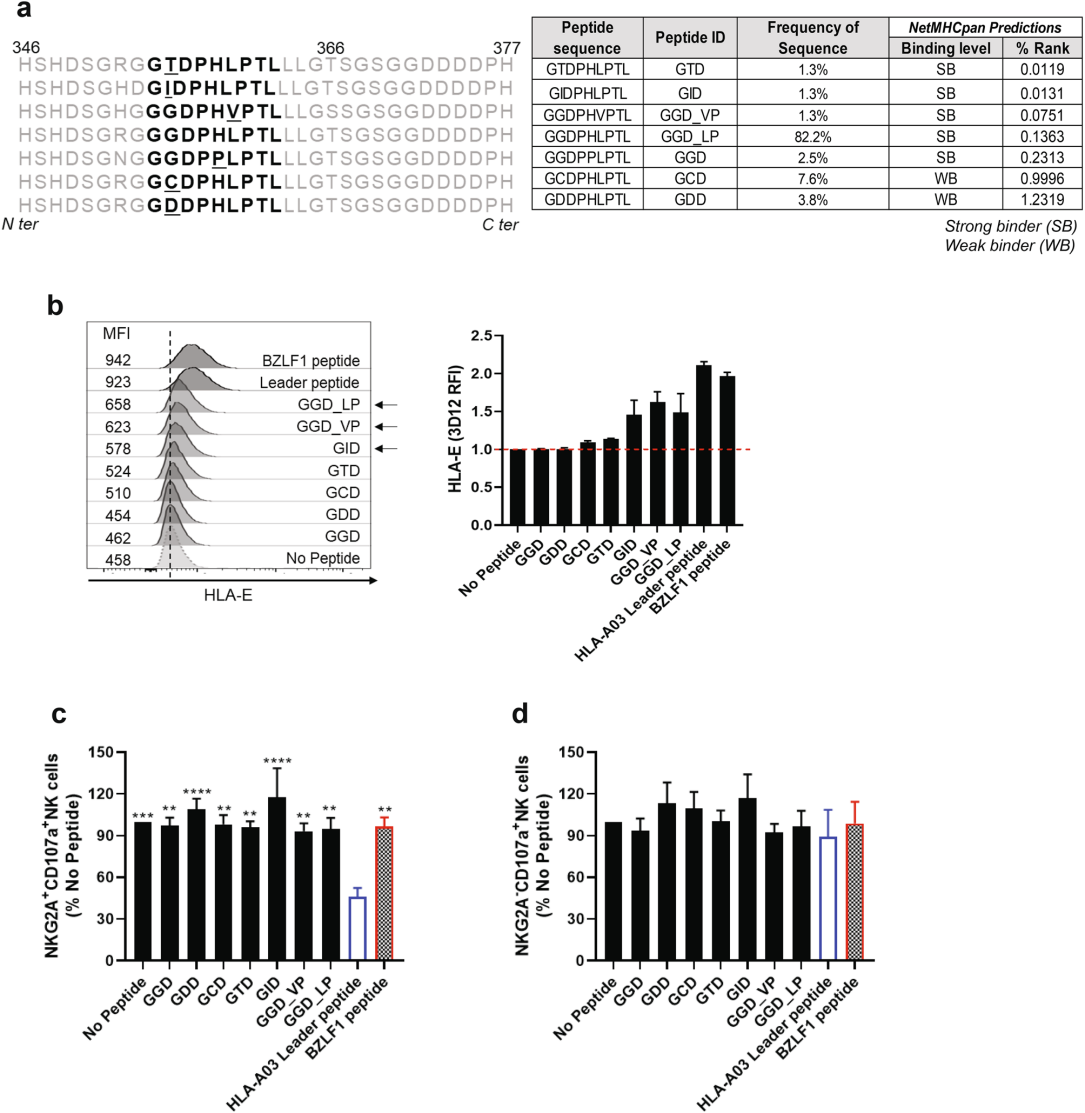
EBV peptide sequence variations alter NKG2A + NK cell effector function (**a**) Naturally occurring sequence variation (highlighted in bold) in the LMP1 transmembrane domain detected in 79 pediatric transplant recipients. (**b**) HLA-E expression by cells pulsed with LMP1 peptide variants. Numbers represent MFI. Peptides that did bind to HLA-E are shown with the black arrows. On the left panel, HLA-E mean fluorescence intensity (MFI) values are shown as relative mean fluorescence intensity (RFI) as compared to HLA-E expression in absence of peptide (No Peptide). The dashed red line marks the absence of HLA-E upregulation. (**c**) NKG2A + NK cell degranulation against target cells loaded with LMP1 peptide variants. Peptides were individually tested using 721.174 cells as target cells (black bars). HLA-A03 and BLZF1 peptides (white filled and checkered bars, respectively) were used as negative (inhibition) and positive (no inhibition) controls. All values have been normalized to “No Peptide.” Data shown is representative of 5 donors. One-way ANOVA was used to compare the leader peptide pulsed condition to others. **p < 0.01, *** p < 0.001 and ****p < 0.0001. (**d**) Comparison of NKG2A-NK cell degranulation against target cells loaded with EBV peptides. The same controls were used as in panel C and all values have been normalized to “No Peptide”. One-way ANOVA test showed no significance differences between the leader peptide pulsed condition and others.

EBV is associated with several malignancies including B cell lymphomas, especially in pediatric recipients of cellular or solid-organ transplants. Analysis of LMP1 sequencing data of a cohort of pediatric transplant recipients revealed amino acid variations within the sequence of Peptide 12 (GGDPHLPTL). We sequenced amino acid 192 to 386 of the cytoplasmic domain of LMP1 of EBV from blood samples obtained from 79 pediatric transplant recipients. The exact nine amino sequence (GGDPHLPTL) that corresponded to Peptide 12 which was found in our in silico analysis, was present in 82.2% (65/79) of the samples (Fig. 4a). The remaining 14 samples displayed one of six variations of the sequence. We synthesized all seven sequences detected in the patient samples and tested them in vitro using assays previously described. We assessed whether these sequence variations influence HLA-E stabilization and NKG2A + NK cell function. Two peptides [GGDPHVPTL (GGD_VP) and GIDPHLPTL (GID)], which were identified in 2.6% of the samples, demonstrated stabilization of HLA-E comparable to the original peptide, while three other peptides [GGDPPLPT (GGD), GCDPHLPTL (GCD), GDDPHLPTL(GDD) and GTDPHLPTL(GTD) ], which were detected in 15.2% of samples, did not stabilize HLA-E (Fig. 4b). The two HLA-E binding peptide variants and the original Peptide 12 (shown by the black arrows in Fig. 4b) were subsequently used to investigate their impact on NK cell function (Fig. 4c,d and Fig. S5a-b). Although GGD, GDD, GCD and GTD peptides did not bind to HLA-E, they were included in the degranulation assays and showed the effect of non-binders on NK cell degranulation. Peptide 12 (GGD_LP) binds HLA-E (Figs. 2a and 4b) and significantly increased degranulation of NKG2A + NK cells as compared to the HLA-A03 leader peptide. (Fig. 3b). The GGD_VP and GID variations did also induce degranulation of NKG2A + NK cells.

As expected, there was no difference in levels of degranulation with NKG2A- cells with all tested peptides since there is no inhibitory signaling through NKG2A and HLA-E (Fig. 4d and Fig. S5b). Overall, we observed that some, but not all, variations of the GGDPHLPTL peptide did bind to HLA-E, thus stabilizing HLA-E expression. Similar to the GGDPHLPTL peptide, they also prevented NKG2A + NK cell inhibition. Further studies are necessary to determine if there is an association between LMP1 sequence variations and NK cell mediated killing of B cells latently infected with EBV.

### EBV peptides render target cells susceptible to NKG2A + NK cell killing

To investigate the ability of primary NK cells to kill peptide-loaded target cells, we performed cytotoxicity assays. As HLA-E also recognizes NKG2C which is an activating receptor^30,36^, sorted NKG2A + 2C- NK cells were used to confirm that NKG2A + NK cells mediate killing of peptide pulsed target cells. NK cells were enriched by magnetic separation (purity range of 78.3 – 96.6%) prior to sorting for NKG2A + 2C- cells (purity range of 95.9 – 98.1% NKG2A + NKG2C-) (Fig. S6a-b). As CMV infection increases the frequency of NKG2C expressing NK cells^37^, we selected CMV-negative donors for our experiments to ensure a higher yield of NKG2A + 2C- NK cells. Cell Trace Violet labelling of the target cells allowed us to separate our unlabeled effectors from the target cells (Fig. S6c).

**Figure 5:**
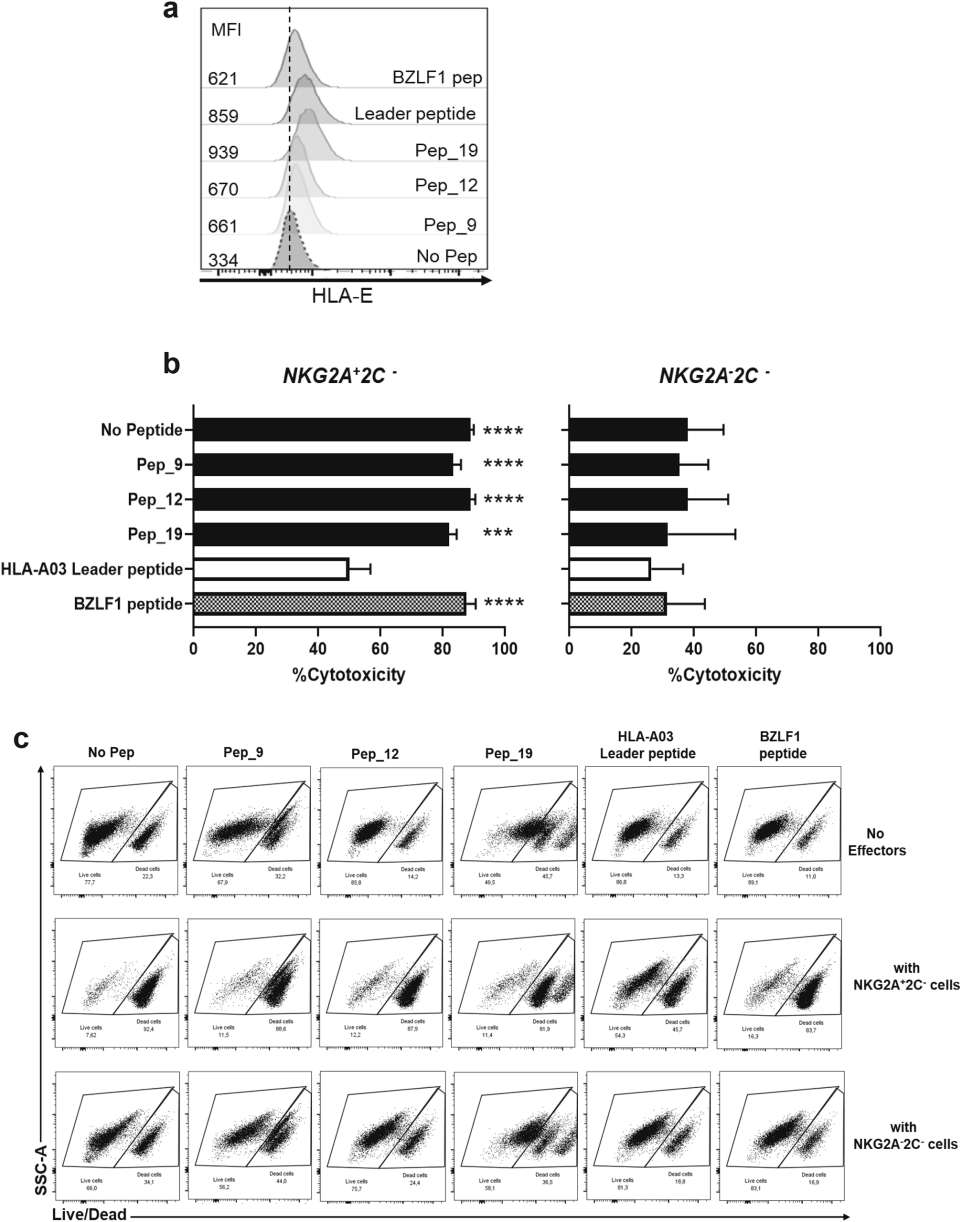
EBV peptides induce specific killing by NK cells expressing NKG2A. (**a**) HLA-E stabilization after peptide loading. (**b**) Comparison of target cell killing when loaded with EBV peptides at E:T ratios of 3:1. HLA-A03 leader peptide and BLZF1 peptide were used as controls. The percentage of cytotoxicity after co-culture of 721.174 cells loaded with EBV peptides and sorted NKG2A + /-NKG2C- cells was calculated with the following formula: [(Control − Test)/Control] × 100 = % of Cytotoxicity. Data shown is representative of 3 donors. One-way ANOVA comparing HLA-A03 leader peptide with all different conditions (***p = 0.0001, ****p < 0.0001). No significant differences were observed in the NKG2A-2C-cells. (**c**) Gating strategy allowing the distinction between live cells (Live/Dead negative) and dead cells (Live/Dead negative).

Three non-inhibitory peptides, Peptides 9, 12 and 19, which bound HLA-E (Fig. 2a) and allowed NKG2A + NK cell degranulation (Fig. 3a) were tested in the cytotoxicity assay. HLA-E upregulation was assessed in parallel with the killing assay (Fig. 5a). As expected, the HLA-A03 leader peptide inhibited NKG2A + NK cell killing as compared to the BZLF1 peptide control (Fig. 5b left). Cytotoxicity exceeded 80% ± SEM for all three peptides. This indicates that despite binding HLA-E, these peptides do not inhibit killing mediated by NKG2A + NKG2C- NK cells. NKG2A-NKG2C- NK cells did not mediate any specific cytotoxicity when we compared the different conditions (Figs. 5b, right and 5c).

### EBV peptide sequences impact the recognition of NKG2A/CD94 receptor and lead to the absence of inhibition despite the presence of HLA-E at the cell surface

**Figure 6:**
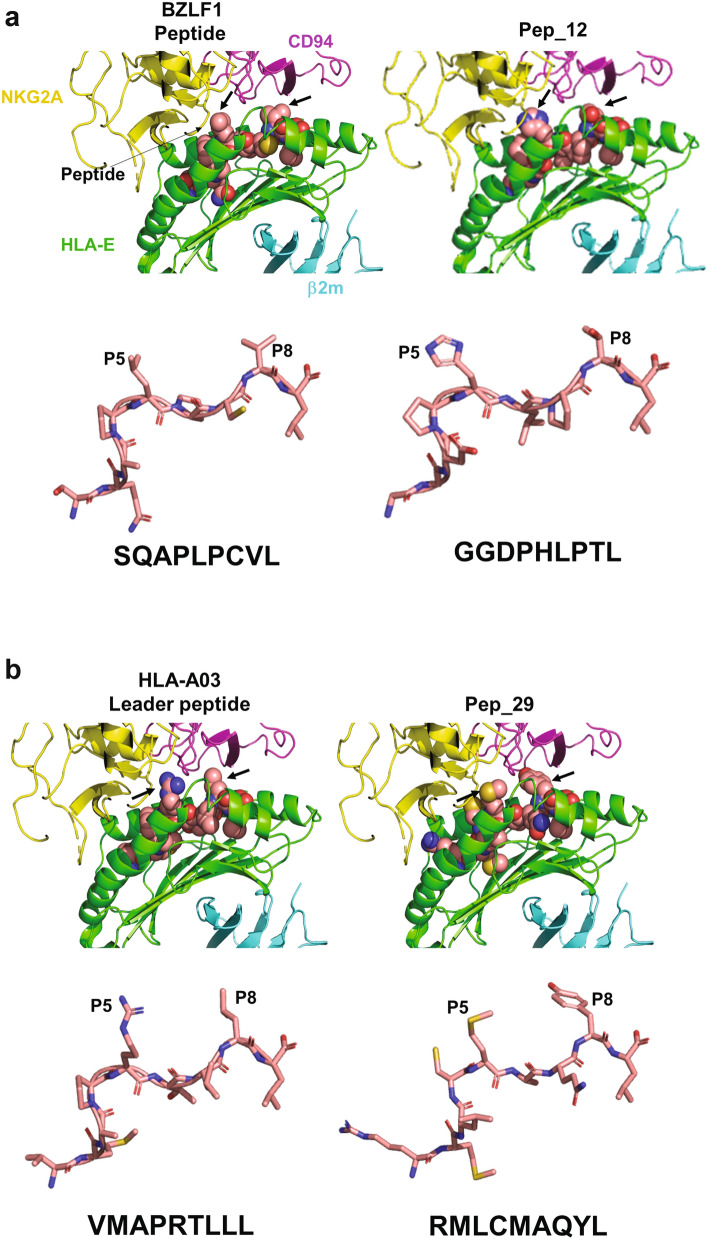
Comparison of presentation of the peptide/HLA-E complex and its recognition by NKG2A/CD94 receptor complex (PDB ID:3CDG). Modelling of individual peptides with P5 and P8 residues identified by black arrows in the HLA-E peptide complex and labelled in the peptide structure. (**a**) Panel of non-inhibitory peptides (BZLF1 peptide and Peptide 12). (**b**) Panel of inhibitory peptides (HLA-A03 leader peptide and Peptide 29).

Previous binding studies have shown that the two most critical peptide contact points for human NKG2x/CD94 with HLA-E peptide complex are positions 5 (p5) with an invariant arginine (R), and 8 (p8) with a variable hydrophobic residue in class I leaders^31,38,39^. Kaiser et al. reported the crystal structure of a complex between NKG2A/CD94 and HLA-E^40^ and we utilized this crystal structure (PBD ID: 3CDG) to model our peptides [Peptide 12, non-inhibitory (Fig. 6a) and Peptide 29, inhibitory (Fig. 6b)] in order to appreciate their conformation in the peptide groove and how their conformation influences binding to the NKG2A/CD94 receptor. We also modelled the two peptide controls used in this study [BZLF1 peptide (Fig. 6a) and HLA-A03 leader peptide (Fig. 6b)]. Different conformations for inhibitory peptides and non-inhibitory peptides were observed, with a wider distance between p5 and p8 for the non-inhibitory peptides (residues pointed by the arrows). These observations suggest that a non-inhibitory peptide will not favor the heterodimerization of NKG2A with its co-receptor CD94, thus impairing the function of the receptor complex. These generated models suggest that EBV peptide sequences presented by HLA-E impact recognition of the NKG2A/CD94 receptor, leading to the absence of inhibition despite the presence of HLA-E at the cell surface.

## Discussion

We identified several HLA-E epitopes from EBV latent proteins, and we provided functional evidence demonstrating that a subset of these EBV-derived peptides presented by HLA-E can prevent inhibition of primary NK cell resulting in killing activity. Our previous work has shown that NKG2A + NK cells can recognize and kill latently infected EBV + B cells^27^. This was striking considering that EBV + B cells expressed MHC class I, specifically HLA-E, and that the presence of peptide-loaded HLA-E on cells is canonically thought to serve as an inhibitory signal for NK cells through NKG2A. We demonstrate that EBV peptides derived from latent proteins can negate inhibition of NKG2A thus allowing NK-mediated killing.

Viral peptides presented by HLA-E have been shown to modulate immune responses. HLA-E can bind and present viral peptides such as AISPRTLNA from HIV (HIVp24_14–22_) and induce HLA-E upregulation on CD4 expressing T cells, resulting in an increase of NK cell inhibition through NKG2A^41,42^. Further, the peptide SQAPLPCVL from EBV BZLF1 protein residues 39–47 (EBVbzlf_39–47_) has been shown to bind to HLA-E to prevent NK cell inhibition^35,43^. Thus, the contribution of specific viral peptides in the modulation of the immune response is important. Recent studies have demonstrated that blocking the interaction between HLA-E and its receptor NKG2A can enhance NKG2A-bearing NK cell or T cell responses^44,45^. Using reverse immunology, we identified peptides that bind to HLA-E and stabilize HLA-E expression at the surface of a TAP-deficient cell. Functional assays further support that a subset of these EBV peptides do allow NKG2A + NK cell mediated effector functions against EBV peptide presenting cells. Additionally, sequence analysis of the LMP1 peptide GGDPHLPTL from a cohort of pediatric transplant recipients at risk for EBV-associated disorders demonstrated that the majority encode a peptide capable of binding HLA-E yet allowing NK cell killing of infected cells. However, some subjects do express LMP1 with sequence variations that do not allow binding to HLA-E, suggesting clearance of latently infected EBV + B cells could be hindered. Additional studies are necessary to address the relationship between sequence variations in LMP1 with EBV disease.

Studies have demonstrated that the NKG2A and NKG2C receptors recognize mostly overlapping, but partially distinct, epitopes on HLA-E^36^. For our cytotoxicity assays, we sorted NKG2A + NKG2C- cells to eliminate any involvement of NKG2C in response to peptide loaded target cells. Our findings indicate that NKG2A + NKG2C- cells did kill target cells that present non-inhibitory peptides. Results confirmed that the presented peptide is interfering with the role of NKG2A receptor in mediating NK cell inhibition.

Peptide sequences from EBV latent proteins may be impairing the recognition of the NKG2A/CD94 heterodimer by impacting key residues at positions 5 (p5) and 8 (p8) based on modeling using PyMOL. Indeed, the structure of NKG2A/CD94 in complex with HLA-E loaded with a leader sequence has been determined^40,46^ and has revealed that NKG2A and CD94 interact with the α1 and α2 helices of the peptide-binding region of HLA-E, respectively, with charge complementarity^47^. Most importantly, crystal structures also revealed that CD94 mainly recognized HLA-E and the peptide, as compared to NKG2A. Investigations showed that arginine (R) at p5 and phenylalanine (F) at p8 are key residues within the peptide sequence that contribute to the binding. Changing the nature of these residues can therefore alter the interaction between the ligand and its receptor. The importance of these residues is reinforced by the fact that they are commonly conserved in most of the leader peptide sequences and their replacement by unsuitable residues leads to the dramatic reduction or abrogation of the binding of the ligand to its receptor. NKG2A/CD94 binds HLA-E by a lock-and-key mechanism and the binding restrains both the entire complex. The CD94 component is mainly responsible for the interaction, while the NKG2A element of the receptor complex transmits the signal intracellularly.

Recent studies in both human and mouse have shown that blocking NKG2A enhances both T and NK cell effector function leading to an efficient anti-tumor effect. André et al. described a novel checkpoint inhibitory mechanism by targeting NKG2A + NK and T cells in combination with anti-EGFR or anti-PD-x antibodies and thus improving patients’ immunotherapy treatment^44^. Besides, improved NK cell effector function against tumors was also shown in mice with blocked expression of NKG2A^45^. In the context of viral infections such as HIV, elevated levels of HLA-E/NKG2A interactions have been shown to reduce HIV-infected target cell clearance by NK cells^48^, suggesting that blocking the interaction will unleash NK cell killing and emphasizing a role for this in novel therapeutics. Utilizing peptides from EBV latent cycle proteins represent a more specific target to alter the HLA-E/NKG2A axis. This approach may limit potential non-specific effects and could be particularly important in immunocompromised patients susceptible to infectious complications.

Using a reductionist system with cells lacking normal levels of HLA, we demonstrated that peptides derived from EBV latent proteins can bind to the HLA-E molecule and alter NK cell functions. Our data provide the first evidence that peptides derived from EBV latent cycle proteins can impair the recognition of NKG2A despite being presented by HLA-E, leading to NKG2A + NK cell activation, and suggest that blocking the NKG2A-HLA-E axis would be an effective strategy to eliminate EBV + B cell lymphomas.

## Materials and methods

### In silico analysis

The protein sequences of the EBV latent proteins LMP1, LMP2, EBNA-1, EBNA-2, EBNA-3A, EBNA-3B and EBNA-3C from the B95.8 laboratory strain of EBV were retrieved using the UniProtKB database (https://www.uniprot.org/). FASTA sequences are listed in the supplementary information (Fig. S1). To predict the binding of peptides to HLA-E, we used the NetMHC pan server (https://www.cbs.dtu.dk/services/NetMHCpan-3.0/), which uses artificial neural network (ANNs) for the predictions^49–51^. HLA-E has two alleles (HLA-E*0101 and HLA-E*0103) which differ by one amino acid at position 107, and the HLA-E*0101 allele was used for this study. All sequence alignments and SeqLogo representation were generated using the GibbsCluster program (https://www.cbs.dtu.dk/services/GibbsCluster/).^52,53^ GibbsCluster is a server for unsupervised alignment and clustering of peptide sequences. The program clusters a list of peptide sequences into meaningful groups.

### Cells lines, PBMC and cell culture

721.174 cells are transporter-associated with antigen processing (TAP) deficient cells^54^ and permit exogenous peptide loading. PBMC from twelve healthy donors, after informed consent, were used as source of NK cells (effector cells) during degranulation and cytotoxicity assays. All human experimental protocols were approved by the Panel On Medical Human Subjects – Stanford University.

Cells were all cultured in R10 medium [RPMI 1640 medium (Lonza, Basel, Switzerland) supplemented with 1% penicillin/streptomycin (Invitrogen, Carlsbad, USA) and 10% FBS (Sigma Aldrich, Saint Louis, USA)]. All cells were maintained in culture at 37 °C, 5% CO_2_ and in humidified atmosphere.

### Peptides

HLA-E restricted peptides from EBV latent cycle proteins were synthetized and purchased from GenScript (New Jersey, USA). High Performance Liquid Chromatography (HPLC) and Mass Spectrometry (MS) confirmed their identities and the purity was greater than 95%. Peptides were dissolved in DMSO at 20 mM and stored in − 80 °C.

### Peptide stabilization

To determine if EBV peptides stabilize HLA-E on the cell surface, 721.174 cells (2 × 10^5^/well) or autologous LCL were incubated overnight at 26 °C, 5% CO_2_ in R10 alone or in R10 medium containing 0–200 μM of the specified peptide. After incubation with the peptides, cells were washed twice with wash buffer (PBS 1X/ 1% BSA/ 0.1% NaN_3_) and re-suspended in blocking buffer (wash buffer + 10% human AB serum) then incubated for 30 min at 4 °C. Cells were then incubated at 4 °C for 30 min with an anti-HLA-E antibody conjugated to PE (clone 3D12, BioLegend, San Diego, USA). After two washes, cells were re-suspended in fixing buffer [PBS 1X/1% PFA (Santa Cruz, Santa Cruz, USA)] and analyzed on a BD LSRII Flow Cytometer using BD Diva Software (BD Biosciences, San Jose, USA). Results were analyzed using FlowJo software and Mean Fluorescence Intensity (MFI) values plotted using GraphPad Prism, version 7.04.

### Degranulation assay

Human PBMCs were isolated from the blood of four healthy donors using Hypaque-Ficoll (GE Healthcare, Chicago, USA) density centrifugation. PBMCs (3 × 10^5^/well) were stimulated overnight with 1 ng/mL recombinant human IL-15 (R&D Systems, Minneapolis, USA). Peptide-pulsed 721.174 target cells or autologous LCL were prepared as for the stabilization assays. Target cells were resuspended with PBMCs at an effector-to-target (E:T) ratio of 5:1 in fresh R10 medium containing peptide and anti-CD107a-efluor-660 antibody (Clone eBioH4A3, eBioscience, Santa Clara, USA). Cells were incubated for 1 h at 26 °C, 6 µg/mL Golgi-Stop (BD Biosciences, San Jose, USA) was added, and the cells were incubated for a further 4 hr at 26 °C. Cells were washed in wash buffer (PBS 1X/ 1% BSA/ 0.1% NaN_3_) and blocked with blocking buffer (10% human serum in wash buffer) for 30 min and then stained with the following antibodies: anti-CD3-PerCP (clone UCHT1, BioLegend, San Diego, USA), anti-human CD56- FITC (Clone HCD56, BioLegend, San Diego, USA), anti-human NKG2A-PE (Clone Z199, Beckman Coulter, Brea, USA) and anti-human NKG2C-AF700 (clone #134,591, R&D systems, Minneapolis, USA). Cells were fixed in 1% PFA and staining were analyzed on a BD LSRII Flow Cytometer with BD Diva Software (BD Biosciences, San Jose, USA).

### Killing assay and peptide stabilization

NKG2A + and NKG2A − NK cells were purified from NK cells after NK cells isolation using an NK Isolation Kit (Miltenyi) and cultured in RPMI complete with 200 U/ml IL-2 overnight. NK cells were then stained with NKG2A-PE (Clone Z199, Beckman Coulter, Brea, USA), NKG2C-APC (clone REA205, Miltenyi, San Diego, USA) CD3-PerCP-Cy5.5 (clone UCHT1, BioLegend, San Diego, USA), CD56-FITC (Clone HCD56, BioLegend, San Diego, USA), and Fixable Viability Dye eFluor 780 (eBioscience, Santa Clara, USA) and sorted for live CD3 − CD56 + and NKG2A ± on a BD FACSAria (BD Biosciences, San Jose, USA). Sorted cells were rested in RPMI complete with 200 U/ml IL-2 (NIH Reagent Program) overnight at 37 °C, 5% CO_2_ before the start of the killing assay. Peptide pulsed target cells were counted and then seeded in a 96 U-bottom well plate with 200 µM peptide in a final volume of 100 µL. The next day, target cells were labeled with 5 µM CellTrace Violet (Invitrogen, Carlsbad, USA) for 20 min at 26 °C, then washed twice with RPMI complete. Target cells (1 × 10^5^) were cocultured with 3 × 10^5^ sorted NKG2A + or NKG2A − NK cells for 5 h in at 26 °C at a 3:1 E:T ratio. At the end of the co-culture, cells were stained for viability using Fixable Viability Dye eFluor 780 [(eF780) at the manufacturer’s recommended concentration for 20 min at 4 °C. Cells were then washed and fixed before analysis. The percentage of cytotoxicity after co-culture of 721.174 cells loaded with EBV peptides and NK cells was calculated with the following formula: [(Control – Test)/Control] × 100 = % of Cytotoxicity.

eF780^−^ Control = n live cells/total target cells in absence of effector cells (spontaneous death).

eF780^−^ Test = n live cells/total target cells in presence of effector cells.

Additionally, peptide loaded target cells were also analyzed for their HLA-E expression as described above (see “Peptide stabilization” section).

### LMP1 sequencing

#### Samples

Patient samples were obtained from the NIH-funded Clinical Trials in Organ Transplant in Children (CTOTC-06) study. Whole blood from 79 pediatric recipients of kidney, liver, heart and intestinal allografts were obtained following Institutional Review Board (IRB) approval and all methods were performed in accordance with the relevant guidelines and regulations. For participants under the age of 18 years, informed consent have been obtained from a parent and/or legal guardian. The mean age at transplant was 6.7yrs (range < 1-21 yr) and the subjects were 54% male and 46% female. Whole blood (500 μl) was added to 1.3 µl of RNA-Later (Thermofisher, Waltham, MA) and stored at -80° C. Genomic DNA was isolated from thawed blood using the Purelink Genomic DNA Mini Kit (Invitrogen, Carlsbad, CA, USA) and the protocol was modified to account for presence of RNA-Later. These modifications included increasing the amounts of Proteinase K and RNAse A added to 100 µl, as well as the amounts of Lysis/Binding Buffer and 100% ethanol during lysis and loading steps to 600 μl. Quality and purity of DNA isolates was assessed through A260/A280 and A260/A230 ratios. DNA was stored in Ultra-pure DNAse-free water and stored at -20° C.

#### PCR amplification

Nested polymerase chain reaction (nested-PCR) was performed to amplify the C-terminal region of LMP1. Two rounds of PCR were completed for each sample, with a third round being completed if no amplification was detected following the second round of nested PCR. Reactions were carried out in a total volume of 50 µl, with all rounds containing 75 mM Tris–HCl, 20 mM (NH4)2SO4, 0.01% Tween 20, 2.5 mM MgCl2, 2.5 U *Taq* DNA Polymerase (Thermofisher, Waltham, MA, USA), 0.3 mM dNTP (Invitrogen, Carlsbad, CA, USA), and 0.5 μM each primer. Primer sequences are displayed in Table 1. The first round contained 250 ng genomic DNA and the second round contained 2.5 µl of PCR product from round 1. If a third round was necessary, PCR product from round 2 was diluted 1:250 and 2.5 μL was added to round 3 master mix. The PCR was performed with an initial denaturation step at 95° C for 10 min. This was followed by 35 cycles of denaturation at 95° C for 30 s, annealing at 55° C for 30 s, extension at 72° C for 1 min and one elongation. The nested-PCR products were visualized by electrophoresis with a 2% agarose gel, stained with SYBR Safe DNA Gel Stain (Thermofisher, Waltham, MA, USA). Successful amplifications were PCR purified with QIAquick PCR Purification Kit (Qiagen, Hilden, Germany) and stored in Ultra-pure DNase-free water at -20° C.

**Table 1:** Primers for LMP1 sequencing.

Primers	Sequences
LMP1 nesting 1F	(5′ TCC TAG ACC TCA TCC TGC TCA T 3′)
LMP1 nesting 1R	(5′ CAA GCC TAT GAC ATG GTA ATG 3′)
LMP1 nesting 2F	(5′ GTC ATA GTA GCT TAG CTG AA 3′)
LMP1 nesting 2R	(5′ TGG ACA ACG ACA CAG T 3′)
LMP1 nesting 3F	(5′ AGT CAT AGT AGC TTA GCT GAA 3′)
LMP1 nesting 3R	(5′ CAG TGA TGA ACA CCA CCA CG 3′)

#### Cloning

Purified PCR products were cloned into a TOPO cloning vector using a TOPO TA Cloning Kit for Sequencing (Invitrogen, Carlsbad, CA, USA). Following cloning, 2 μL of TOPO reaction were added to DH5a competent cells and incubated for 30 min on ice. Cells were immediately heat shocked at 42 °C for 30 s, before adding 250 μL S.O.C. media and incubating for one hour at 37° C with shaking. 100 μL of transformation was plated overnight on LB Agar plates with ampicillin. Following overnight incubation, 12 colonies per sample were inoculated in liquid LB broth with ampicillin and grown up overnight. Tubes were then spun down and plasmids were isolated with a PureLink Quick Plasmid Miniprep Kit (Invitrogen, Carlsbad, CA, USA). Isolated plasmids were submitted to Elim Biopharm (Hayward, CA) for sequencing with M13 reverse primers. Sequences were aligned and analyzed with Geneious 10.2.5 software.

#### Peptides

GGDPHLPT peptide variants [GGDPPLPT (GGD), GCDPHLPTL (GCD), GDDPHLPTL(GDD) GGDPHVPTL (GGD_VP) and GIDPHLPTL (GID)] were synthetized and purchased from GenScript (New Jersey, USA). High Performance Liquid Chromatography (HPLC) and Mass Spectrometry (MS) confirmed their identities and the purity was greater than 95%. Peptides were dissolved in DMSO at 20 mM and stored in − 80 °C.

### Structure visualization

To model the ligand and receptor structure, a Python script which launches a built-in PyMOL plug-in was used. We utilized the backbone of a published structure (PBD ID: 3CDG) from Petrie et al^46^. Peptide sequences have been changed using the mutagenesis tool to model each epitope. These structures did undergo energy minimization, allowing for a mild global relaxation of the peptide.

### Statistical analysis

All graphs and statistical analyses were performed using GraphPad Prism, version 7.04 (GraphPad Software).

## Supplementary information

Supplementary Information.
