# Supplementary material for: Epstein–Barr virus peptides derived from latent cycle proteins alter NKG2A + NK cell effector function
Source: Sci Rep. 2020 Nov 17;10:19973. doi: 10.1038/s41598-020-76344-3 (PMC7673117; doi:10.1038/s41598-020-76344-3)
Supplement: Supplementary file 1 — Supplementary Information. [file 41598_2020_76344_MOESM1_ESM.pdf]

Epstein-Barr virus peptides derived from latent cycle proteins alter NKG2A+ NK cell effector function.

Berenice Mbiribindi[1], Josselyn K. Pena[1], Matthew P. Arvedson[1], Claudia Romero Moreno[1], Sarah R. McCarthy[1], Olivia L. Hatton[2], Carlos O. Esquivel[1], Olivia M. Martinez[1] and Sheri M. Krams[1*]

[1]Division of Abdominal Transplantation, Department of Surgery, Stanford University School of Medicine, Stanford, California, USA

[2]Department of Molecular Biology, Colorado College, Colorado Springs, Colorado, USA

***Corresponding author**

Sheri M. Krams, Ph.D.
Professor of Surgery/Division of Abdominal Transplant
Stanford University School of Medicine
MSLS P313
Stanford, CA 94305-5492
650-498-6246
650-498-6250 (FAX)
smkrams@stanford.edu

a

>LMP1

MEHDLERGPPGPRRPPRGPPLSSSLGLALLLLLLALLFWLYIVMSDWTGGALLVLYSFALML
IIIILIIFIFRRDLLCPLGALCILLLMITLLLIALWNLHGQALFLGIVLFIFGCLLVLGIWI
YLLEMLWRLGATIWQLLAFFLAFFLDLILLIIALYLQQNWWTLLVDLLWLLLFLAILIWMYY
HGQRHSDEHHHDDSLPHPQQATDDSGHESDSNSNEGRHHLLVSGAGDGPPLCSQNLGAPGGG
PDNGPQDPDNTDDNGPQDPDNTDDNGPHDPLPQDPDNTDDNGPQDPDNTDDNGPHDPLPHSP
SDSAGNDGGPPQLTEEVENKGGDQGPPLMTDGGGGHSHDSGHGGGDPHLPTLLLGSSGSGGD
DDDPHGPVQLSYYD

>LMP2

MGSLEMVPMGAGPPSPGGDPDGYDGGNNSQYPSASGSSGNTPTPPNDEERESNEEPPPPYED
PYWGNGDRHSDYQPLGTQDQSLYLGLQHDGNDGLPPPPYSPRDDSSQHIYEEAGRGSMNPVC
LPVIVAPYLFWLAAIAASCFTASVSTVVTATGLALSLLLLAAVASSYAAAQRKLLTPVTVLT
AVVTFFAICLTWRIEDPPFNSLLFALLAAAGGLQGIYVLVMLVLLILAYRRRWRRLTVCGGI
MFLACVLVLIVDAVLQLSPLLGAVTVVSMTLLLLAFVLWLSSPGGLGTLGAALLTLAAALAL
LASLILGTLNLTTMFLLMLLWTLVVLLICSSCSSCPLSKILLARLFLYALALLLLASALIAG
GSILQTNFKSLSSTEFIPNLFCMLLLIVAGILFILAILTEWGSGNRTYGPVFMCLGGLLTMV
AGAVWLTVMSNTLLSAWILTAGFLIFLIGFALFGVIRCCRYCCYYCLTLESEERPPTPYRNT
V

>EBNA1

MSDEGPGTGPGNGLGEKGDTSGPEGSGGSGPQRRGDNHGRGRGRGRGRGGGRPGAPGGSGS
GPRHRDGVRRPQKRPSCIGCKGTHGGTGAGAGAGGAGAGGAGAGGGAGAGGGAGGAGGA
GAGGGAGAGGGAGGAGGAGAGGGAGAGGGAGGAGAGGGAGGAGAGGGAGAGGGAGGAGA
GGGAGGAGGAGAGGGAGAGGAGGAGGAGAGGAGAGGGAGGAGGAGAGGAGAGGAGAGGAGAG
GAGGAGAGGAGGAGAGGAGGAGAGGGAGGAGAGGGAGGAGAGGAGGAGAGGAGGAGAGGAGG
AGAGGGAGAGGAGAGGGGRGRGGSGGRGRGGSGGRGRGGSGGRRGRGRERARGGSRERARGR
GRGRGEKRPRSPSSQSSSSGSPPRRPPPGRRPFFHPVGEADYFEYHQEGGPDGEPDVPPGAI
EQGPADDPGEGPSTGPRGQGDGGRRKKGGWFGKHRGQGGSNPKFENIAEGLRALLARSHVER
TTDEGTWVAGVFVYGGSKTSLYNLRRGTALAIPQCRLTPLSRLPFGMAPGPGPQPGPLRESI
VCYFMVFLQTHIFAEVLKDAIKDLVMTKPAPTCNIRVTVCSFDDGVDLPPWFPPMVEGAAAE
GDDGDDGDEGGDGDEGEEGQE

>EBNA2

MPTFYLALHGGQTYHLIVDTDSLGNPSLSVIPSNPYQEQLSDTPLIPLTIFVGENTGVPPPL
PPPPPPPPPPPPPPPPPPPPPPPPPPSPPPPPPPPPPPQRRDAWTQEPSPLDRDPLGYDVGH
GPLASAMRMLWMANYIVRQSRGDRGLILPQGPQTAPQARLVQPHVPPLRPTAPTILSPLSQP
RLTPPQPLMMPPRPTPPTPLPPATLTVPPRPTRPTTLPPTPLLTVLQRPTELQPTPSPPRMH
LPVLHVPDQSMHPLTHQSTPNDPDSPEPRSPTVFYNIPPMPLPPSQLPPPAAPAQPPPGVIN
DQQLHHLPSGPPWWPPICDPPQPSKTQGQSRGQSRGRGRGRGRGRGKGKSRDKQRKPGGPWR
PEPNTSSPSMPELSPVLGLHQGQGAGDSPTPGPSNAAPVCRNSHTATPNVSPIHEPESHNSP
EAPILFPDDWYPPSIDPADLDESWDYIFETTESPSSDEDYVEGPSKRPRPSIQ

>EBNA3A

MDKDRPGPPALDDNMEEEVPSTSVVQEQVSAGDWENVLIELSDSSSEKEAEDAHLEPAQKGT
KRKRVDHDAGGSAPARPMLPPQPDLPGREAILRRFPLDLRTLLQAIGAAATRIDTRAIDQFF
GSQISNTEMYIMYAMAIRQAIRDRRRNPASRRDQAKWRLQTLAAGWPMGYQAYSSWMYSYTD
HQTTPTFVHLQATLGCTGGRRCHVTFSAGTFKLPRCTPGDRQWLYVQSSVGNIVQSCNPRYS
IFFDYMAIHRSLTKIWEEVLTPDQRVSFMEFLGFLQRTDLSYIKSFVSDALGTTSIQTPWID
DNPSTETAQAWNAGFLRGRAYGIDLLRTEGEHVEGATGETREESEDTESDGDDEDLPCIVSR

GGPKVKRPPIFIRRLHRLLLMRAGKRTEQGKEVLEKARGSTYGTPRPPVPKPRPEVPQSDET
ATSHGSAQVPEPPTIHLAAQGMAYPLHEQHGMAPCPVAQAPPTPLPPVSPGDQLPGVFSDGR
VACAPVPAPAGPIVRPWEPSLTQAAGQAFAPVRPQHMPVEPVPVPTVALERPVYPKPVRPAP
PKIAMQGPGETSGIRRARERWRPAPWTPNPPRSPSQMSVRDRLARLRAEAQVKQASVEVQPP
QLTQVSPQQPMEGPLVPEQQMFPGAPFSQVADVVRAPGVPAMQPQYFDLPLIQPISQGAPVA
PLRASMGPVPPVPATQPQYFDIPLTEPINQGASAAHFLPQQPMEGPLVPEQWMFPGAALSQS
VRPGVAQSQYFDLPLTQPINHGAPAAHFLHQPPMEGPWVPEQWMFQGAPPSQGTDVVQHQLD
ALGYTLHGLNHPGVPVSPAVNQYHLSQAAFGLPIDEDESGEGSDTSEPCEALDLSIHGRPCP
QAPEWPVQEEGGQDATEVLDLSIHGRPRPRTPEWPVQGEGGQNVTGPETRRVVVSAVVHMCQ
DDEFPDLQDPPDEA

>EBNA3B
MKKAWLSRAQQADAGGASGSEDPPDYGDQGNVTQVGSEPISPEIGPFELSAASEDDPQSGPV
EENLDAAAREEEEPHEQEHNGGDDPLDVHTRQPRFVDVNPTQAPVIQLVHAVYDSMLQSDLR
PLGSLFLEQNLNIEEFIWMCMTVRHRCQAIRKKPLPIVKQRRWKLLSSCRSWRMGYRTHNLK
VNSFESGGDNVHPVLVTATLGCDEGTRHATTYSAGIVQIPRISDQNQKIETAFLMARRARSL
SAERYTLFFDLVSSGNTLYAIWIGLGTKNRVSFIEFVGWLCKKDHTHIREWFRQCTGRPKAA
KPWLRAHPVAIPYDDPLTNEEIDLAYARGQAMNIEAPRLPDDPIIVEDDDESEEIEAESDEE
EDKSGMESLKNIPQTLPYNPTVYGRPAVFDRKSDAKSTKKCRAIVTDFSVIKAIEEEHRKKK
AARTEQPRATPESQAPTVVLQRPPTQQEPGPVGPLSVQARLEPWQPLPGPQVTAVLLHEESM
QGVQVHGSMLDLLEKDDEVMEQRVMATLLPPVPQQPRAGRRGPCVFTGDLGIESDEPASTEP
VHDQLLPAPGPDPLEIQPLTSPTTSQLSSSAPSCAQTPWPVVQPSQTPDDPTKQSRPPETAA
PRQWPMPLRPIPMRPLRMQPIPFNHPVGPTPHQTPQVEITPYKPTWAQIGHIPYQPTPTGPA
TMLLRQWAPATMQTPPRAPTPMSPPEVPPVPRQRPRGAPTPTPPPQVPPVPRQRPRGAPTPT
PPPQVLPTPMQLALRAPAGQQGPTKQILRQLLTGGVKKGRPSLKLQAALERQAAAGWQPSPG
SGTSDKIVQAPIFYPPVLQPIQVMGQGGSPTAMAASAVTQAPTEYTRERRGVGPMPPTDIPP
SKRAKIEAYTEPEMPHGGASHSPVVILENVGQGQQQTLECGGTAKQERDMLGLGDIAVSSPS
SSETSNDE

>EBNA3C
MESFEGQGDSRQSPDNERGDNVQTTGEHDQDPGPGPPSSGASERLVPEESYSRDQQPWGQSR
GDENRGWMQRIRRRRRRRAALSGHLLDTEDNVPPWLPPHDITPYTARNIRDAACRAVKQSHL
QALSNLILDSGLDTQHILCFVMAARQRLQDIRRGPLVAEGGVGWRHWLLTSPSQSWPMGYRT
ATLRTLTPVPNRVGADSIMLTATFGCQNAARTLNTFSATVWTPPHAGPREQERYAREAEVRF
LRGKWQRRYRRIYDLIELCGSLHHIWQNLLQTEENLLDFVRFMGVMSSCNNPAVNYWFHKTI
GNFKPYYPWNAPPNENPYHARRGIKEHVIQNAFRKAQIQGLSMLATGGEPRGDATSETSSDE
DTGRQGSDVELESSDDELPYIDPNMEPVQQRPVMFVSRVPAKKPRKLPWPTPKTHPVKRTNV
KTSDRSDKAEAQSTPERPGPSEQSSVTVEPAHPTPVEMPMVILHQPPPVPKPVPVKPTPPPS
RRRRGACVVYDDDVIEVIDVETTEDSSSVSQPNKPHRKHQDGFQRSGRRQKRAAPPTVSPSD
TGPPAVGPPAAGPPAAGPPAAGPPAAGPPAAGPPAAGPRILAPLSAGPPAAGPHIVTPPSAR
PRIMAPPVVRMFMRERQLPQSTGRKPQCFWEMRAGREITQMQQEPSSHLQSATQPTTPRPSW
APSVCALSVMDAGKAQPIESSHLSSMSPTQPISHEEQPRYEDPDAPLDLSLHPDVAAQPAPQ
APYQGYQEPPAPQAPYQGYQEPPPPQAPYQGYQEPPAHGLQSSSYPGYAGPWTPRSQHPCYR
HPWAPWSQDPVHGHTQGPWDPRAPHLPPQWDGSAGHGQDQVSQFPHLQSETGPPRLQLSLVP
LVSSSAPSWSSPQPRAPIRPIPTRFPPPPMPLQDSMAVGCDSSGTACPSMPFASDYSQGAFT
PLDINATTPKRPRVEESSHGPARCSQATAEAQEILSDNSEISVFPKDAKQTDYDASTESELD

b

Peptide ID	Peptide Sequence	HLA Restriction	Peptide Source
Pep_1	YLQQNWWTL	HLA-E*0101	LMP1
Pep_2	VLYSFALML	HLA-E*0101	LMP1
Pep_3	MSNWTGGAL	HLA-E*0101	LMP1
Pep_4	SSIGLALLL	HLA-E*0101	LMP1
Pep_5	LQQNWWTLL	HLA-E*0101	LMP1
Pep_6	SSSIGLALL	HLA-E*0101	LMP1
Pep_7	LSSSIGLAL	HLA-E*0101	LMP1
Pep_8	LGATIWQLL	HLA-E*0101	LMP1
Pep_9	VLFIFGCLL	HLA-E*0101	LMP1
Pep_10	IFIFRRDLL	HLA-E*0101	LMP1
Pep_11	LVDLLWLLL	HLA-E*0101	LMP1
Pep_12	GGDPHLPTL	HLA-E*0101	LMP1
Pep_13	SSPGGLGTL	HLA-E*0101	LMP2
Pep_14	IVAPYLFWL	HLA-E*0101	LMP2
Pep_15	TLNLTTMFL	HLA-E*0101	LMP2
Pep_16	TLAAALALL	HLA-E*0101	LMP2
Pep_17	VVSATGLAL	HLA-E*0101	LMP2
Pep_18	VVSMTLLLL	HLA-E*0101	LMP2
Pep_19	RLFLYALAL	HLA-E*0101	LMP2
Pep_20	ILQTNFKSL	HLA-E*0101	LMP2
Pep_21	STEFIPHLF	HLA-E*0101	LMP2
Pep_22	CSSCSSCPL	HLA-E*0101	LMP2
Pep_23	FIPHLFCML	HLA-E*0101	LMP2
Pep_24	SSTEFIPHL	HLA-E*0101	LMP2
Pep_25	AVLQLSPLL	HLA-E*0101	LMP2
Pep_26	VFMSLGGLL	HLA-E*0101	LMP2
Pep_27	LTPLSRLPF	HLA-E*0101	EBNA1
Pep_28	SMPQLSPVV	HLA-E*0101	EBNA2
Pep_29	RMLCMAQYL	HLA-E*0101	EBNA2
Pep_30	TLPQPRIPL	HLA-E*0101	EBNA2
Pep_31	RQEAPIILL	HLA-E*0101	EBNA2
Pep_32	FTPVPMVAL	HLA-E*0101	EBNA2
Pep_33	SYSIPSMTL	HLA-E*0101	EBNA2
Pep_34	LTLGHQLSL	HLA-E*0101	EBNA2
Pep_35	VTPSATPDI	HLA-E*0101	EBNA2
Pep_36	AQAWNAGFL	HLA-E*0101	EBNA3 A
Pep_37	AMQPQYFDL	HLA-E*0101	EBNA3 A
Pep_38	ILRRFPLDL	HLA-E*0101	EBNA3 A
Pep_39	HGAPAAHFL	HLA-E*0101	EBNA3 A
Pep_40	VAQAPPTPL	HLA-E*0101	EBNA3 A
Pep_41	RMGYRTQTL	HLA-E*0101	EBNA3 B
Pep_42	LMARRARSL	HLA-E*0101	EBNA3 B

Pep_43	LSPYRTWRM	HLA-E*0101	EBNA3 B
Pep_44	LSAERYTLF	HLA-E*0101	EBNA3 B
Pep_45	AMQLSPRAL	HLA-E*0101	EBNA3 B
Pep_46	LAYARGLAM	HLA-E*0101	EBNA3 B
Pep_47	AMSIEAARL	HLA-E*0101	EBNA3 B
Pep_48	PMPLRPIPL	HLA-E*0101	EBNA3 B
Pep_49	SLSAERYTL	HLA-E*0101	EBNA3 B
Pep_50	SAERYTLFF	HLA-E*0101	EBNA3 B
Pep_51	AQLEPWQPL	HLA-E*0101	EBNA3 B
Pep_52	IMAPPVVRM	HLA-E*0101	EBNA3 C
Pep_53	RFPPPPMPL	HLA-E*0101	EBNA3 C
Pep_54	RMFMRERQL	HLA-E*0101	EBNA3 C
Pep_55	RAALSGHLL	HLA-E*0101	EBNA3 C
Pep_56	RLQLSSVPL	HLA-E*0101	EBNA3 C
Pep_57	MAPPVVRMF	HLA-E*0101	EBNA3 C
Pep_58	LQALSNLIL	HLA-E*0101	EBNA3 C
Pep_59	YSQGAFTPL	HLA-E*0101	EBNA3 C
Pep_60	TSPSQSWPM	HLA-E*0101	EBNA3 C
Pep_61	GVGWRHWLL	HLA-E*0101	EBNA3 C
HLA-A03 Leader peptide	VMAPRTLLL	HLA-E*0101	HLA-03
BZLF1 peptide	SQAPLPCVL	HLA-E*0101	EBV BZLF1

Figure S1: *In silico* analysis of HLA-E binders derived from EBV latent proteins, Related to Figure 1. (a) Protein sequences retrieved from UniProtKB database. (b) List of 61 HLA-E*0101 strong binders generated from NetMHCpan analysis. The list includes peptides control HLA-A03 leader peptide and BZLF1 peptide.

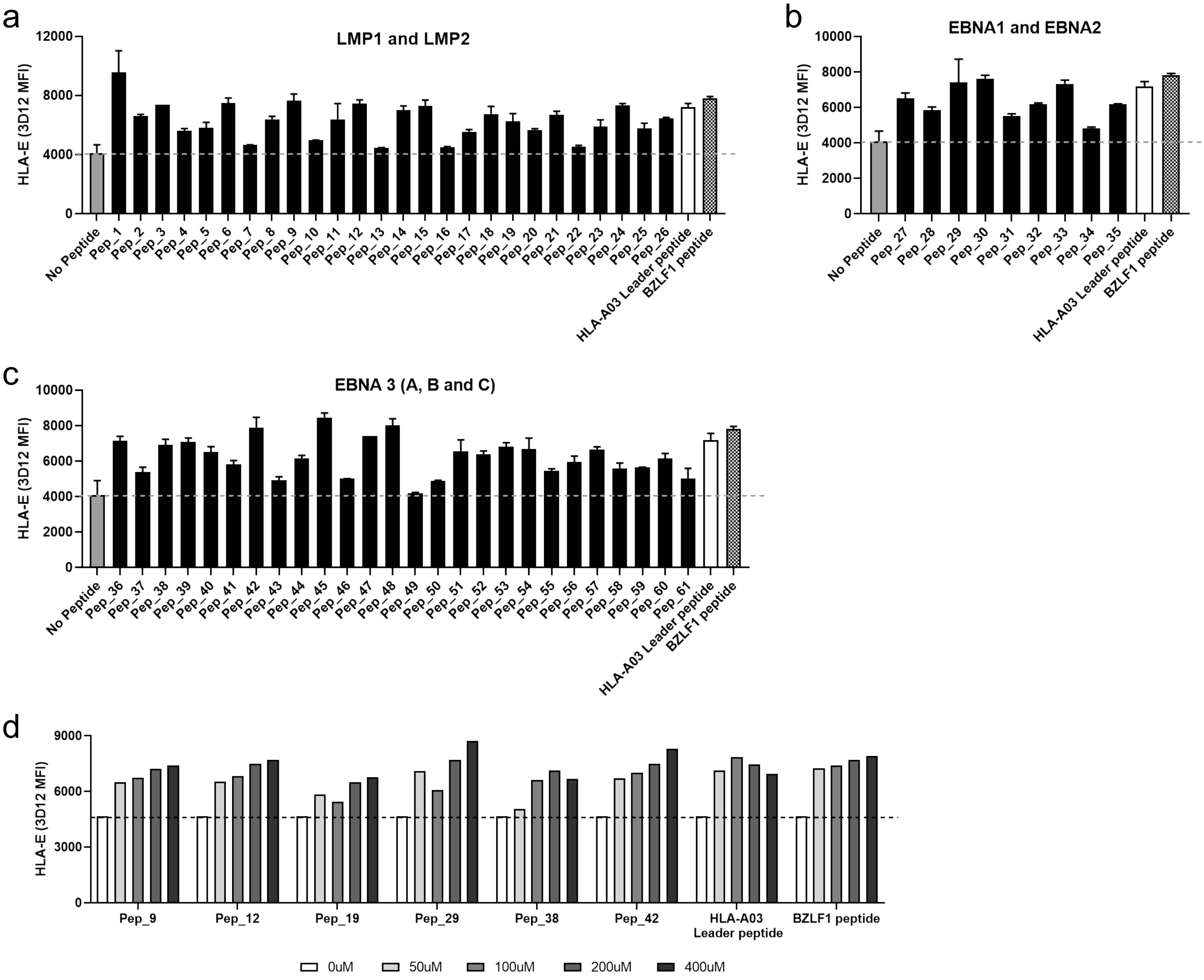

Figure S2: MFI of HLA-E on the surface of 721.174 cells pulsed with EBV peptides, Related to Figure 2. (a) LMP1 and LMP2, (b) EBNA-1 and ENBA-2, (c) EBNA-3A, -3B and -3C derived peptides were pulsed onto target cells. HLA-E expression was assessed the following day. Peptides that did bind to HLA-E induced an upregulation of HLA-E (MFI values) surface expression and were compared to the background expression in absence of peptide (No Peptide). Endogenous peptide, HLA-A03 leader peptide (white filled bar) and viral peptide, BZLF1 (checkered bar) derived from EBV were used as positive controls. The dashed grey line represents the cut-off set by No Peptide. Data shown is representative of the mean of two representative experiments. (d) Initial peptide titration using various concentrations of peptide (presentation of selcted peptides as an example).

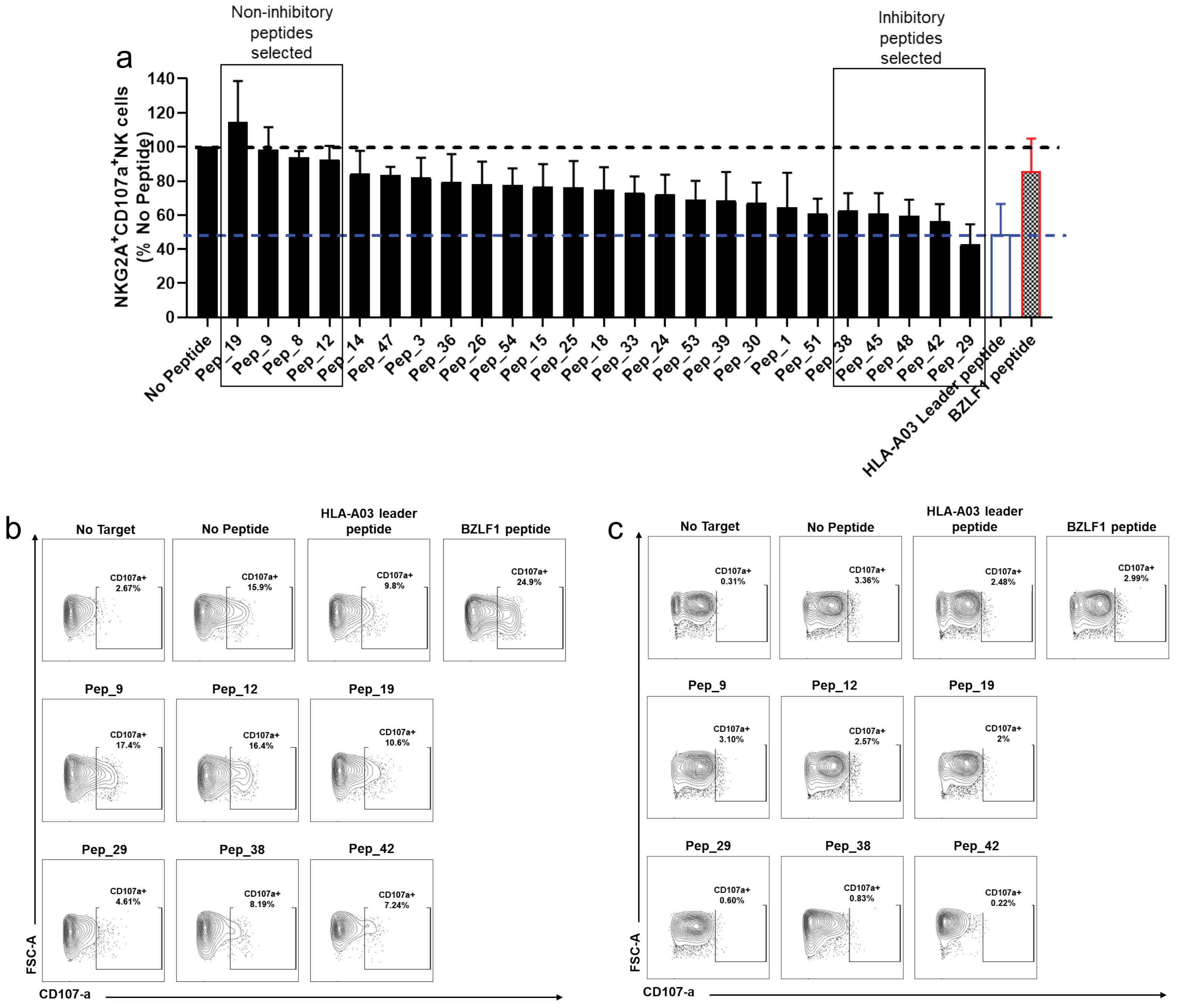

Figure S3: Degranulation assays and gating strategy, Related to Figure 3. (a) Degranulation of NKG2A+ NK cells against target cells loaded with HLA-E binders. (b) Degranulation of NKG2A+ NK cells toward selected peptides. (c) Degranulation of NKG2A- NK cells toward selected peptides.

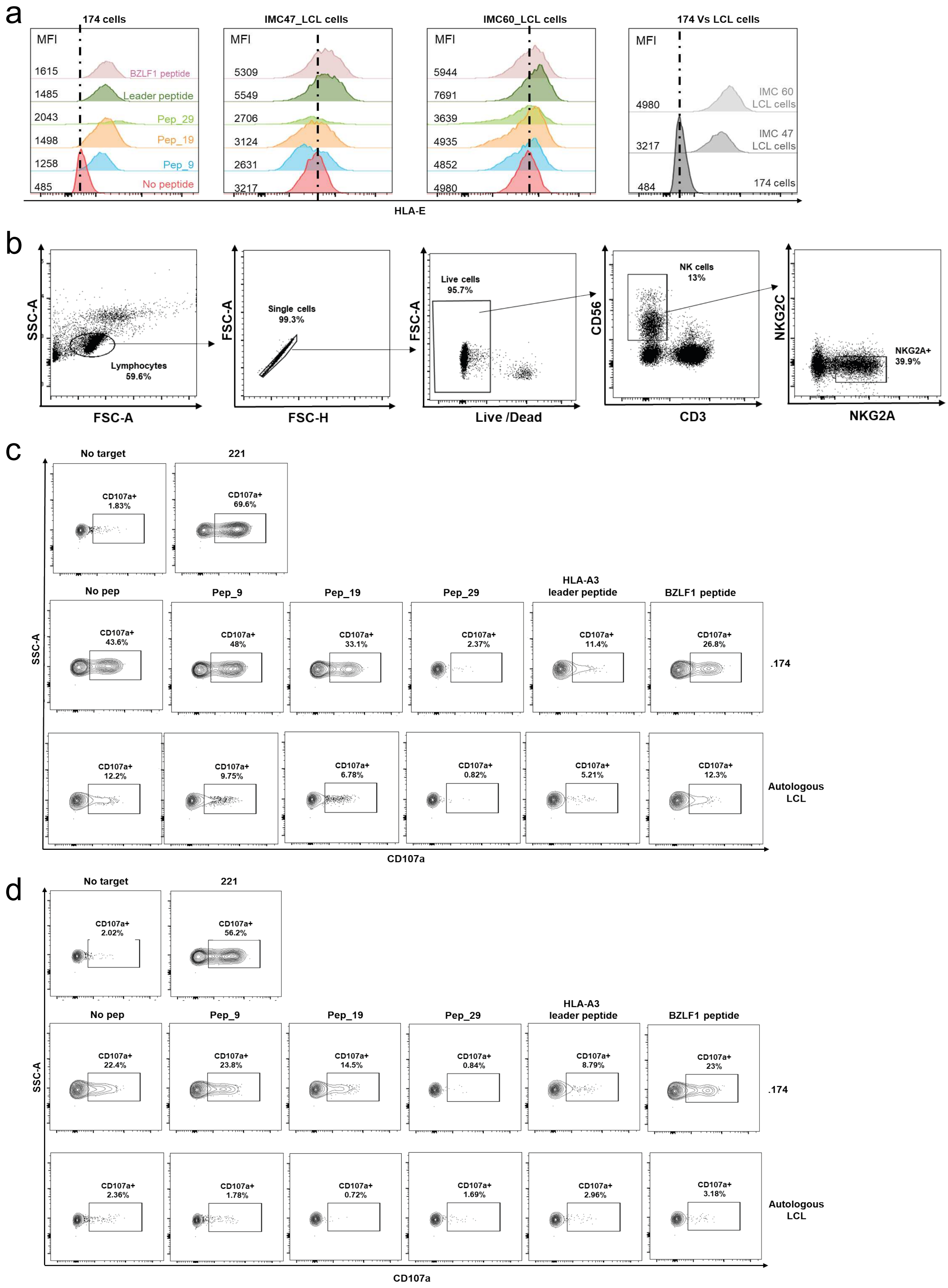

Figure S4: Comparison of NKG2A+ NK cells degranulation after co-culture with 721.174 cells or autologous LCL. (a) HLA-E expression by 721.174 cells or LCL (IMC47 and IMC60) pulsed with EBV peptides derived from latent proteins. Endogenous peptide, HLA-A03 leader peptide and viral peptide, BZLF1 from EBV peptide were used as positive controls for HLA-E up regulation at the cell surface. Numbers correspondent to MFI. (b) Gating strategy used to analyze CD107a expression from NKG2A+ NK cells. After gating on live cells and NK cells (CD3-CD56+), NKG2C cells were excluded and only NKG2A+ cells were selected for the evaluation of CD107a expression. (c) IMC47 donor NKG2A+ NK cell degranulation against target cells loaded with EBV derived peptides. Peptides were individually tested using 721.174 cells as target cells (middle plots) or autologous LCL (lower plots). HLA-A03 and BZLF1 peptides respectively, were used as negative (inhibition) and positive (no inhibition) controls. (d) IMC60 donor NKG2A+ NK cell degranulation against target cells loaded with EBV derived peptides.

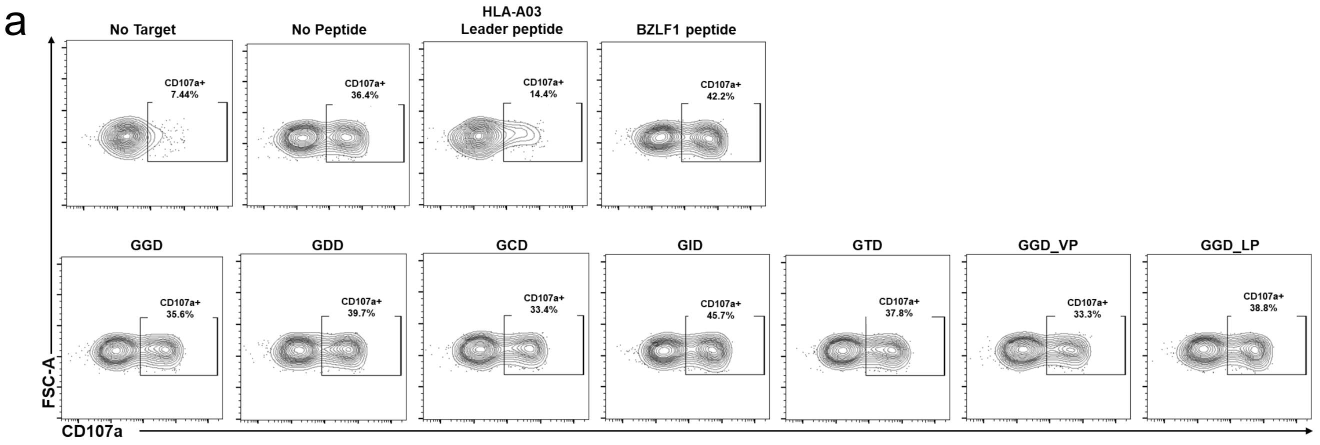

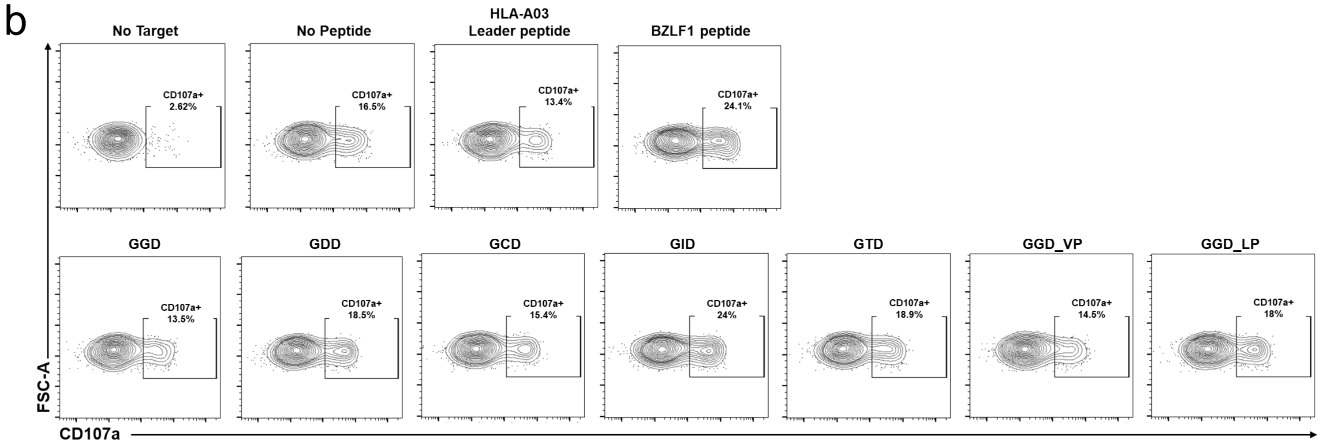

Figure S5: Degranulation assays gating strategy, Related to Figure 4. (a) Degranulation of NKG2A+ NK cells. (b) Degranulation of NKG2A- NK cells.

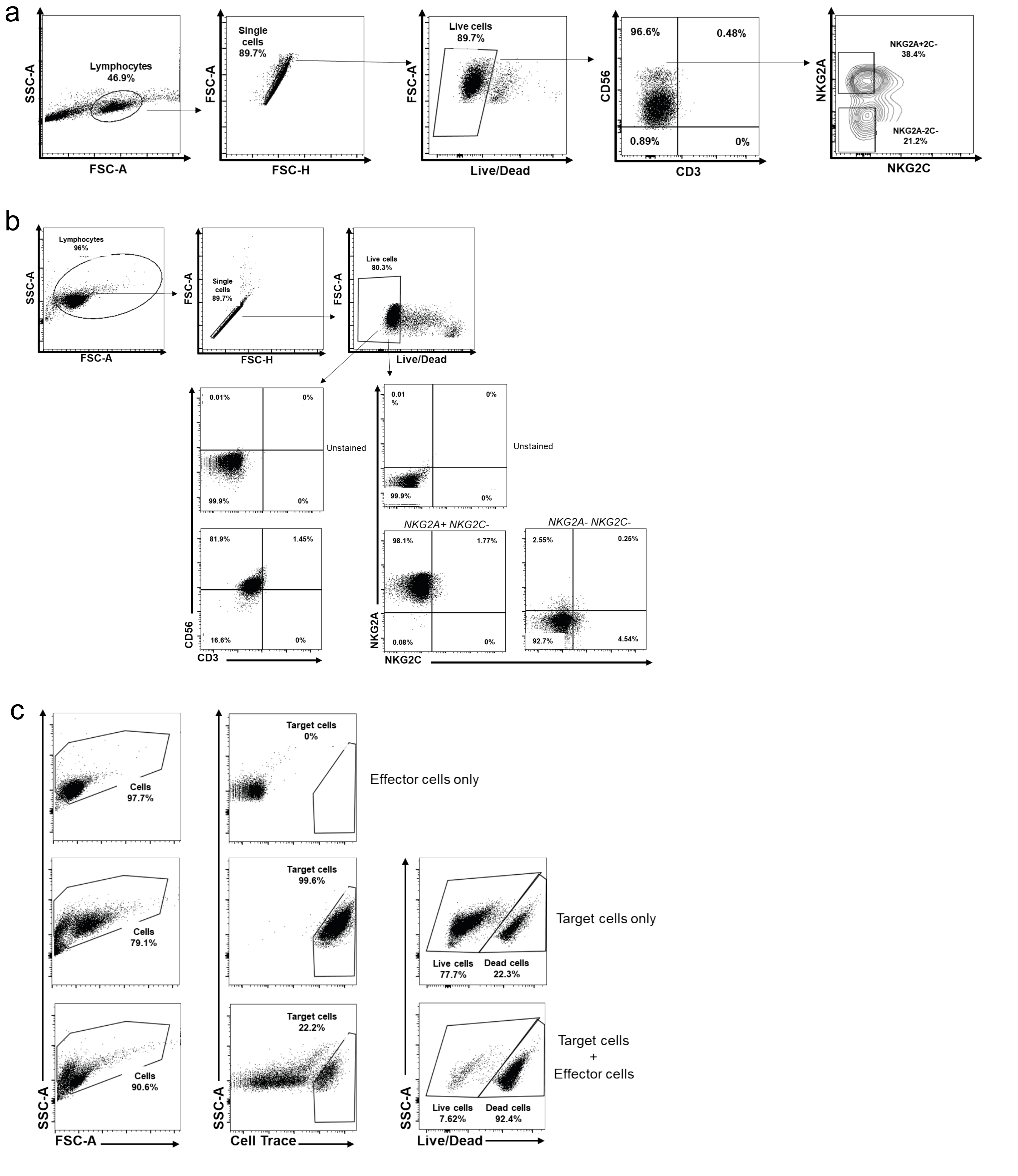

Figure S6: Sorting strategy, post sorting assessment and killing assay gating strategy, Related to Figure 5. (a) Freshly isolated NK cells from PBMC were stained for CD3, CD56, NKG2A and NKG2C. NKG2A+NKG2C- as well as NKG2A-NKG2C- were sorted using a stringent gating strategy. (b) Sorted cells were assessed for population purity using the same panel as for the sorting. (c) Gating strategy and identification of effectors cells, targets cells and live or dead cells within target cells.
